# Knowledge, attitude, and preventive behaviors related to HIV/AIDS and sexually transmitted infections among Myanmar migrants in Chiang Mai province, Thailand

**DOI:** 10.3389/fpubh.2024.1478592

**Published:** 2024-11-27

**Authors:** Kyaw Soe Thant, Aksara Thongprachum, Sineenart Chautrakarn, Pannawich Chantaklang, Suwat Chariyalertsak

**Affiliations:** ^1^Faculty of Public Health, Chiang Mai University, Chiang Mai, Thailand; ^2^Nakornping Hospital, Chiang Mai, Thailand

**Keywords:** Myanmar migrant, HIV/AIDS, preventive behaviors, STI, Thailand

## Abstract

**Introduction:**

Despite progress in controlling HIV/AIDS and STIs, these health challenges persist, with 39.9 million people living with HIV in 2023 and more than 350 million affected by STIs annually. Thailand is a major migrant hub in Southeast Asia. This study investigated the factors influencing preventive behaviors related to HIV/AIDS and STIs among Myanmar migrants in Chiang Mai province, Thailand.

**Methods:**

A cross-sectional study was conducted in 2023 among 424 Myanmar migrants aged 18–45 years. Participants were selected through convenience sampling from a local hospital and community gathering locations in Chiang Mai province. Multiple logistic regression analysis was used to identify factors influencing preventive behaviors.

**Results:**

The participants’ average age was 29.92 years; 56.8% were married, and 67.5% had completed primary or lower education. The average monthly income was <10,000 THB (47.6%). Most participants had a good knowledge level of HIV (63.2%), while 80% had poor knowledge of STIs. Among the sexually active participants (*n* = 274), 91.2% had good preventive behavior. The multivariate analysis indicated that migrants working in non-construction or day labor roles were less likely to practice preventive behaviors (aOR = 0.210, 95% CI: 0.046–0.972, *p* = 0.046). Additionally, migrants who had lived in Chiang Mai for more than 10 years were less likely to engage in preventive behaviors (aOR = 0.067, 95% CI: 0.010–0.465, *p* = 0.006). There was a statistically significant association between preventive behaviors and a positive attitude, with an aOR of 4.575 (95% CI: 1.226–17.073, *p* = 0.024).

**Conclusion:**

Low STI knowledge and negative attitudes toward condom use were found in this study. Culturally relevant and sensitive interventions and effective sexual health education are needed.

## 1 Introduction

Despite three decades of progress in managing human immunodeficiency virus (HIV), it remains a major global health challenge. By 2023, 39.9 million people were living with HIV (PLWHA) worldwide, approximately 630,000 deaths due to HIV-related causes and only 77% of PLWHA received antiretroviral therapy (1). Globally, the burden of sexually transmitted infections (STIs) is significant, with approximately 1 million infections daily from four curable STIs including syphilis (2). In Thailand, an estimated 560,000 people were living with HIV in 2023, with an incidence rate of 0.13 per 1,000 uninfected population (3). In addition, syphilis accounted for the highest number of STI cases by 2021 (4). In Chiang Mai province, the case rate of five common STI infections (Syphilis, Gonorrhea, Chlamydia, Chancroid, and Lymphogranuloma Venereum) was 69.6 per 100,000 population in 2021 (5).

World Health Organization (WHO) aims to end AIDS, viral hepatitis, and STIs by 2030 through coordinated strategies (2). Thailand’s National Strategy to End AIDS: 2017–2030 targets reducing new HIV cases to <1,000 annually, AIDS-related deaths to <4,000 annually, and decreasing discrimination based on HIV or gender by 90% (6). Key populations for the strategy include men who have sex with men, sex workers, people who inject drugs, prisoners, and migrant workers.

Thailand, a major migrant hub in South-East Asia (7), hosts 2,167,937 documented migrant workers as of 2022, primarily from Myanmar, Cambodia, and Laos People’s Democratic Republic (Laos PDR) where 60.9% from Myanmar (7). In Chiang Mai Province, there were 101,377 migrant workers in December 2022, with 99% from Myanmar (8). Migrants, though not categorized as a key population by WHO, are considered as vulnerable group for HIV/AIDS and STI infections (9). The increasing trend of transborder migration heightens the risk of spreading infectious diseases, with an observed increasing rate of HIV transmission in migrant populations worldwide (10). The prevalence rate of HIV among migrant workers in Thailand was 0.2% in 2020 (4). Among the 12 provinces in Thailand with higher migrant populations (4), Chiang Mai had the highest prevalence rate at 1.3%.

Risk factors for HIV/AIDS and syphilis among migrants include limited condom use, having multiple partners, lack of HIV/AIDS knowledge (9, 11–13), and having a sexual partner with an STI (14). Studies highlight reasons for inconsistent condom usage as limited knowledge and poor attitudes toward condoms among migrants (15–18). External migrants face unprotected sex risks due to low-income status, debt, homelessness, and limited access to medical care (19). Mobility increases their vulnerability to STIs (10) and health risks for migrants in terms of susceptibility to and dissemination, which are not unidirectional (20). Challenges in HIV/AIDS care for cross-country migrants include low levels of knowledge on condom use, limited access to testing and counseling services, stigma, and discrimination (21).

A 2011 nationwide hospital-based survey in Thailand revealed that migrant workers faced substantially higher relative risks for sexually transmitted infections, tuberculosis, and malaria compared to Thai workers (22). Existing studies on HIV/AIDS among migrants in Chiang Mai have predominantly focused on sub-populations such as sex workers, people living with HIV/AIDS, and cross-border youths, with limited studies on the general migrant population (17, 18). This study aims to address this gap by investigating knowledge, attitudes, and preventive behaviors related to HIV/AIDS and STI infections among general migrants in Chiang Mai province. This study’s findings could provide insights for public health professionals and policymakers in the region, informing targeted preventive activities against HIV/AIDS and STIs among migrants.

## 2 Materials and methods

### 2.1 Study design and sample

This cross-sectional study was conducted among Myanmar migrant workers in Chiang Mai province, Thailand from October to December 2023. The sample size was calculated for a single proportion of binomial outcomes in an infinite population, using a population proportion of 50%, a 5% level of estimation, and a 95% confidence interval, resulting a minimal sample size of 385. After adding a 10% incomplete data rate, a total of 424 participants were included in the study (23). Adult migrants aged 18 to 45 years, were recruited using convenience sampling from Nakornping Hospital and community gathering settings. The recruitment occurred at the migrant health screening site of Nakornping Hospital, where four trained research assistants, fluent in Tai-Yai and Myanmar, approached individuals seeking health services. To identify migrants, the research assistants conversed with attendees and used a brief screening process to confirm their migrant status. The inclusion criteria were as follows: participants had to be (1) adults aged 18 to 45 years, (2) currently residing in Chiang Mai for over 1 month, (3) fluent in Tai-Yai or Myanmar, and (4) willing to provide informed consent for participation in the survey. Data was collected in a gender-matched approach with participants who consented to the survey. After 6 weeks of recruitment at Nakornping Hospital, administrative changes reduced migrant visits and health screening days. Consequently, data collection was extended to community gathering places, such as monasteries, where Myanmar migrants frequently gather for social gatherings, ensuring that the recruitment remained focused on the target population. The study was approved by the research ethics committees of the Faculty of Public Health, Chiang Mai University (ET041/2023) and Nakornping Hospital (NKP no. 148/66).

### 2.2 Instrument

The survey questionnaire used in the Prevention of HIV/AIDS among Migrant Workers in Thailand 2 program (PHAMIT-2) (24) was adjusted and developed in English. It was validated by three experts with extensive experience in the HIV/AIDS field and translated into Tai-Yai and Myanmar by native speakers. The overall index of item-objective congruence (IOC) was 0.86, indicating strong relevance of the items to the study objectives, as experts provided scores of 1, 0, and − 1 based on their opinions of relevance, neutrality, and irrelevance, respectively. The questionnaire consisted of six parts: (1) socio-demographic data; (2) HIV/AIDS knowledge questions, comprising seven items with responses of “Yes,” “No,” and “Do not Know.” A score of 1 was assigned for correct answers and 0 for incorrect or “Do not Know.” Knowledge levels were categorized as “Good” if the score was ≥4.82 (mean score), otherwise “Poor”; (3) STI knowledge was classified as “Good” if the score was ≥0.76 (mean score) out of 5, otherwise “Poor”; (4) six items on a Likert scale assessed attitudes toward HIV, while four items each assessed attitudes toward condom use and STIs. A total of 14 items on a Likert scale evaluated overall attitudes toward HIV/AIDS, condom use, and preventive behaviors, ranging from 5 (Strongly Agree) to 1 (Strongly Disagree), with negative items reverse-scored. Attitudes were categorized as “Good” if the score exceeded the mean in each domain. For the overall attitude assessment, a score above 51.28 out of 70 (mean score) was categorized as “Good,” otherwise as “Poor”; and (5) questions on sexual behaviors.

In this study, preventive behavior was measured by three criteria among participants who had sexual intercourse: not engaging with a non-regular partner, always using condoms with non-regular partners in the past 12 months, and consistently using condoms with sex workers in the past 6 months. Participants received a score of 1 for each criterion they met. Those with a score of 1 or higher were classified as “Preventive,” while those with a score below 1 were classified as “Un-preventive.” Data were collected through face-to-face interviews and recorded using the Kobo Collect application on smartphones/tablets. It was further tested among 30 Myanmar migrant workers in Chiang Mai Province, showing internal consistency with KR-20 of 0.777 for knowledge scores and Cronbach’s alpha of 0.713 for attitude scores.

### 2.3 Statistical analysis

Data analysis utilized SPSS version 26.0 (SPSS Inc., Chicago, IL, USA). Descriptive statistics (frequency, mean, median, and standard deviation) summarized sociodemographic factors, knowledge, attitudes, and preventive behaviors. Multiple logistic regression identified associations between these factors and preventive behaviors related to HIV/AIDS and STIs.

## 3 Results

### 3.1 Sociodemographic characteristics

Table 1 shows the socio-demographic characteristics of 424 participants, 50% female and 50% male, with a mean age of 29.92 ± 7.9 years. In terms of marital status, 56.4% being married and 25.7% being single. A total of 38.7% had no formal education and 28.8% had primary education. Nearly 32% have been living in Chiang Mai for more than 10 years. More than 63% were construction workers or daily laborers. Nearly half of the participants (47.6%) had an average monthly income of <10,000 THB. The majority (85.8%) had personal identification (ID) status, such as a passport or work permit, allowing them to live and work legally in Chiang Mai Province and 69.8% had health insurance. Only 35.6% had a history of drinking alcohol in the last 3 months.

**Table 1:** Socio-demographic characteristics (*n* = 424).

Characteristics	Male		Female		Total	
	*n* = 212	%	*n* = 212	%	*n* = 424	%
Site						
Hospital	166	78.3	102	48.1	268	63.2
Community	46	21.7	110	51.9	156	36.8
Perceived sexual identity						
Straight man	211	99.5	0	0	211	49.8
Straight woman	0	0	211	99.5	211	49.8
Other	1	0.5	1	0.5	2	0.2
Age group (years)						
18–24	63	29.7	56	26.4	119	28.1
25–34	90	42.5	83	39.2	173	40.8
35–45	59	27.8	73	34.4	132	31.1
Ethnicity						
Shan (Tai Yai)	182	85.8	182	85.8	364	85.5
Lahu	0	0	4	1.9	4	0.9
Pa-O	6	2.8	8	3.8	14	3.3
Kachin	5	2.4	1	0.5	6	1.4
Burmese	8	3.8	5	2.4	13	3.1
Other	11	5.2	12	5.7	23	5.4
Religion						
Buddhism	199	93.9	198	93.4	397	93.6
Christianity	9	4.2	12	5.7	21	5
Islam	1	0.5	1	0.5	2	0.5
Other	3	1.4	1	0.5	4	0.9
Marital status						
Single	64	30.2	45	21.2	109	25.7
Married (spouse present)	132	62.3	107	50.5	239	56.4
Married (living apart)	7	3.3	10	4.7	17	4
Separated/divorced	2	0.9	10	4.7	12	2.8
Other^a^	7	3.3	40	18.8	47	11.1
Education level						
No formal education	95	44.8	69	32.5	164	38.7
Primary school	64	30.2	58	27.4	122	28.8
Middle school	23	10.8	56	26.4	79	18.6
High school	24	11.3	24	11.3	48	11.3
Bachelor’s degree and above	6	2.8	5	2.4	11	2.6
Length of stay in Chiang Mai (years) Median = 8.00 years (Min =0, Max = 34)						
≤1 year	46	21.7	26	12.3	72	17
2–5 years	57	26.9	54	25.5	111	26.2
6–10 years	56	26.4	50	23.6	106	25.0
>10 years	53	25	82	38.7	135	31.8
Occupation						
Construction workers	96	45.3	42	19.8	138	32.5
General workers/daily laborers	73	34.4	59	27.8	132	31.1
Domestic workers	2	0.9	25	11.8	27	6.4
Factory workers	1	0.5	10	4.7	11	2.6
Plantation workers	19	9	25	11.8	44	10.4
Commerce	1	0.5	5	2.4	6	1.4
Housewives	0	0	1	0.5	1	0.2
Others	20	9.4	45	21.2	65	15.3
Average monthly income						
<10,000 THB	87	41	115	54.2	202	47.6
more than 10,000 THB	119	56.1	44	20.8	163	38.4
No response	6	2.8	53	25.0	59	13.9
Personal identification (ID) status in Thailand						
Yes	189	89.2	175	82.5	364	85.8
No	14	6.6	16	7.5	30	7.1
Do not know	6	2.8	0	0	6	1.4
No response	3	1.4	21	9.9	24	5.7
Health insurance status						
No insurance	43	20.3	31	14.6	74	17.5
Have insurance	164	77.4	132	62.3	296	69.8
No response	5	2.4	49	23.1	54	12.7
History of alcohol drinking within the last 3 months						
Yes	126	59.4	25	11.8	151	35.6
No	81	38.2	184	86.8	265	62.5
No response	5	2.4	3	1.4	8	1.9
History of recreational drug use within the last 3 months						
Yes	8	3.8	0	0	8	1.9
No	199	93.9	211	99.5	410	96.7
No response	5	2.4	1	0.5	6	1.4

^a“Others” in Marital status- widowed and living together with partner.^

### 3.2 Knowledge of HIV/AIDS and STI infections

Table 2 presents participant’s knowledge, with 63.2% having “Good” HIV knowledge. The average score was 4.82 (SD = 1.63). Participants had a good level of transmission knowledge and more than 90% of participants correctly responded that HIV can be transmitted through blood transfusion. Regarding HIV prevention knowledge, only 64.2% of participants were aware that HIV can be prevented by condom use.

**Table 2:** Knowledge of HIV/AIDS and STI Infections (*n* = 424).

Questions	Correct answer		Incorrect answer	
	*n*	%	*n*	%
HIV/AIDS transmits through sharing meals	175	41.3	249	58.7
HIV/AIDS transmits through sharing syringes/needles	379	89.4	45	10.6
HIV/AIDS transmits through blood transfusion	387	91.3	37	8.7
HIV/AIDS transmits during pregnancy and delivery	314	74.1	110	25.9
HIV/AIDS transmits during breastfeeding	310	73.1	114	26.9
Antiretroviral drugs prevent transmission during pregnancy	207	48.8	217	51.2
HIV/AIDS can be protected by condoms	272	64.2	152	35.8
**HIV knowledge** **Good 268 (63.2%)** **Poor 156 (36.8%)** **Mean ± SD: 4.82 ± 1.630**				
Have you ever heard about STIS? (*n* = 424)				
Yes (*n* = 98, 23%)				
No (*n* = 326, 77%)				
Name at least one type of STI (*n* = 98)	73	74.5	25	25.5
Name at least one symptom of STIs (*n* = 98)	55	56.1	43	43.9
Name at least one risk of STIs (*n* = 98)	71	72.4	27	27.6
People can prevent STIs by using condoms (*n* = 98)	72	73.5	26	26.5
Name at least one complication of STIs (*n* = 98)	51	52	47	48
**STI knowledge** **Poor 339 (80%)** **Good 85 (20%)** **Mean ± SD: 0.76 ± 1.621**				
			
			

Only 20% of the participants had good STI knowledge with an average score of 0.76 (SD = 1.62). Twenty-three percent of the participants had ever heard of STI infections. Among them, 74.5% could name at least one type of STI and only 56.1% knew any STI symptoms.

### 3.3 Attitude toward HIV/AIDS, condom usage, and STI infections

Table 3 shows migrants’ attitudes toward HIV/AIDS, condom usage and STI infections. The overall mean attitude score was 51.28 (SD = 5.32) with 53.5% of the participants having an overall “Good” attitude. Attitude toward HIV/AIDS was good among participants as 66.5% had an above mean score of 24.25 (SD = 3.66). In contrast, 62.7% had poor attitudes toward condom usage, scoring below the mean of 12.41 (SD = 2.08). Regarding attitude toward STIs, 59.7% had a good attitude with a mean score of 14.61 (SD = 2.10).

**Table 3:** Attitudes toward HIV/AIDS, condom usage, and STI infections (*n* = 424).

I think that…	Strongly agree		Agree		Neutral		Disagree		Strongly disagree		Mean score
	*n*	%	*n*	%	*n*	%	*n*	%	*n*	%	(SD)
Having multiple partners increases the risk of HIV/AIDS	336	79.2	35	8.3	37	8.7	8	1.9	8	1.9	4.61 (0.868)
Drinking alcohol increases the risk of unsafe sex	300	70.8	56	13.2	46	10.8	11	2.6	11	2.6	4.47 (0.967)
A healthy-looking person can transmit HIV/AIDS	298	70.3	44	10.4	48	11.3	20	4.7	14	3.3	4.40 (1.071)
AIDS is a disgusting disease and cannot be treated*	251	59.2	100	23.6	38	8.9	27	6.4	8	1.9	1.68 (1.004)
HIV/AIDS patients will suffer both physically and mentally	321	75.7	59	13.9	32	7.5	3	0.7	9	2.1	4.60 (0.830)
HIV/AIDS patients will have a reduced ability to work	300	70.8	67	15.8	34	8.0	12	2.8	11	2.6	4.49 (0.945)
**Attitude toward HIV infection** **Good attitude 282 (66.5%)** **Poor attitude 142 (33.5%)**											24.25 (3.656)
Needed to use a condom with a regular partner	146	34.4	44	10.4	71	16.7	151	35.6	12	2.8	3.38 (1.346)
Needed to use a condom with a non-regular partner	347	81.9	40	9.4	28	6.6	1	0.2	8	1.9	4.69 (0.767)
Feel embarrassed to buy condoms*	191	45.1	62	14.6	117	27.6	25	5.9	29	6.8	2.15 (1.249)
Wearing a condom reduces sexual feelings*	174	41.1	54	12.7	155	36.6	23	5.4	18	4.2	2.19 (1.158)
**Attitude toward condom usage** **Good attitude 158 (37.3%)** **Poor attitude 266 (62.7%)**											12.41 (2.079)
STIs have a high chance of HIV/AIDS exposure	235	55.4	61	14.4	114	26.9	10	2.4	4	0.9	4.21 (0.980)
STIs are disgusting and immoral*	218	51.4	65	15.3	106	25.1	26	6.1	9	2.1	1.92 (1.094)
Some of STIs are curable	229	54.0	56	13.2	122	28.8	12	2.8	5	1.2	4.16 (1.012)
Partner treatment is important in STIs	264	62.3	50	11.8	99	23.3	3	0.7	8	1.9	4.32 (0.977)
**Attitude toward STIs** **Good attitude 253 (59.7%)** **Poor attitude 171 (40.3%)**											14.61 (2.097)
**Overall HIV/AIDS, STIs, and Condom Attitude level** **Good Attitude 227 (53.5%)** **Poor Attitude 197 (46.3%)** **Mean ± SD: 51.28 ± 5.322**											51.28 (5.322)

*Negative items.

### 3.4 Sexual behaviors related to HIV/AIDS

The majority of the participants (64.6%) reported having had prior sexual experience, with males (75.5%) and females (53.8%). A total of 17.9% did not respond to this question, possibly due to its sensitive nature. Among those with prior sexual experience, 78.4% (*n* = 215) reported having a regular partner, and of these, 77.2% never used condoms with their regular partner.

Of the participants who had prior sexual exposure, 5.8% (*n* = 16) reported having a non-regular partner, with 50% of those having more than one non-regular partner in the last 12 months. Among those with non-regular partners, 43.8% (*n* = 7) reported always using condoms.

Additionally, only 1.5% (*n* = 4) of participants with prior sexual experience reported visiting sex workers in the last 6 months, and of these 75% (*n* = 3) always used condoms (see Supplementary Table 1 for detailed sexual behaviors).

### 3.5 Preventive behaviors

From the total of 424 participants, 274 (64.4%) reported prior sexual experience. Among these, 91.2% reported preventive behaviors, scoring 1 or higher based on the criteria of: not engaging with a non-regular partner, consistently using condoms with non-regular partners in the past 12 months, and always using condoms with sex workers in the past 6 months. After excluding participants who did not respond to questions regarding health insurance status and monthly income, 262 participants were included in the final logistic regression model. Table 4 presents the findings from the multivariate analysis, indicating that migrants in non-construction or day labor roles were less likely to practice preventive behaviors compared to construction workers (aOR = 0.210, 95% CI: 0.046–0.972, *p* = 0.046). Additionally, migrants who had lived in Chiang Mai for more than 10 years were less likely to engage in preventive behaviors (aOR = 0.067, 95% CI: 0.010–0.465, *p* = 0.006). Furthermore, participants with a positive attitude toward HIV/AIDS were 4.5 times more likely to engage in preventive behaviors than those with a negative attitude (aOR = 4.575, 95% CI: 1.226–17.073, *p* = 0.024).

**Table 4:** Univariable and multivariable analysis of factors associated with the preventive behaviors (*n* = 262).

	Univariate		Multivariate	
Factors	Crude OR	*p*-value	Adjusted OR	*p*-value
	(95% CI)		(95% CI)	
Gender				
Male	1		1	
Female	1.558(0.525–4.622)	0.424	1.023 (0.177–5.919)	0.98
Age (years)				
18–24	1	0.578	1	0.373
25–34	0.728 (0.189–2.807)	0.645	0.881 (0.103–7.496)	0.907
35–45	1.378 (0.296–6.406)	0.683	2.571 (0.235–28.145)	0.439
Education level				
Low level	1		1	
High level	0.514 (0.157–1.683)	0.272	0.538 (0.114–2.526)	0.432
Marital status				
Single	1	0.619	1	0.286
Married	1.556 (0.413–5.870)	0.514	4.022 (0.537–30.109)	0.175
Other^a^	0.794 (0.121–5.195)	0.81	1.485 (0.081–27.29)	0.79
Occupation				
Construction workers		0.038*		0.007*
General workers/day laborers	8.48 (1–71.9)	0.05	7.218 (0.716–72.758)	0.094
Other^b^	0.578 (0.195–1.71)	0.322	0.210 (0.046–0.972)	0.046*
Monthly income				
<10,000 THB	1		1	
≥10,000 THB	1.033 (0.376–2.84)	0.95	0.821 (0.219–3.072)	0.77
Insurance status				
No insurance	1		1	
Have insurance	2.01 (0.613–6.584)	0.249	3.223 (0.512–20.299)	0.213
Length of stay in Chiang Mai (years)				
≤5 years	1	0.346	1	0.020*
6–10 years	0.814 (0.211–3.136)	0.765	0.287 (0.041–2.027)	0.211
> 10 years	0.433 (0.132–1.418)	0.167	0.067 (0.010–0.465)	0.006*
ID or document to stay in Thailand				
Yes, have it.	1		1	
Do not have it.	0.837 (0.102–6.834)	0.868	2.364 (0.146–38.294)	0.545
Alcohol drinking (within 3 months)				
Yes, have it.	1		1	
No.	2.201 (0.776–6.247)	0.138	2.359 (0.512–10.865)	0.271
Recreational drugs use (within 3 months)				
Yes, have it.	1		1	
No	4.033 (0.424–38.363)	0.225	3.451 (0.137–86.924)	0.452
HIV knowledge level				
Poor	1		1	
Good	1.245 (0.437–3.546)	0.682	1.387 (0.330–5.826)	0.655
STI knowledge level				
Poor	1		1	
Good	1.248 (0.343–4.536)	0.737	2.154 (0.425–10.916)	0.354
Overall attitude level				
Poor	1		1	
Good	5.016 (1.684–14.94)	0.004*	4.575 (1.226–17.073)	0.024*
Global discrimination toward PLWHA				
No	1		1	
Yes	0.365 (0.047–2.845)	0.336	0.235 (0.021–2.601)	0.238

*Statistically significant at *p* < 0.05. ^a^Other in Marital Status means Separated/Divorced, Widowed, Living together with partner. ^b^Other in Occupation means Domestic Workers, Factory Workers, Plantation Workers, Commerce, Housewife and Other.

## 4 Discussion

The cross-sectional study aimed to examine the factors influencing preventive behaviors related to HIV/AIDS and STIs among Myanmar migrants in Chiang Mai province. Despite a good level of HIV knowledge among participants, most migrants showed a poor level of STI knowledge and a negative attitude toward condom usage.

### 4.1 Knowledge of HIV and STI

Participants demonstrated a good level of HIV knowledge (63.2%), particularly about transmission methods over prevention. Misunderstandings included that HIV/AIDS could be transmitted through sharing meals (58.7%) and unawareness that antiretroviral drugs prevent transmission during pregnancy (51.2%). These misconceptions can increase stigma and delay HIV care. Similar findings in prior studies among Myanmar migrants (12, 25, 26) revealed the ongoing need for accessible health education using simple language and visual aids. Despite good HIV knowledge, its correlation with preventive behaviors was weak, consistent with previous studies (12, 26), indicating the influence of social, cultural, and environmental factors on behaviors. This can be addressed by implementing community-based interventions that promote safe practices, reduce stigma, and create supportive environments for at-risk populations.

The majority (77%) reported limited awareness regarding STIs. This aligns with previous findings from a 2010 nationwide survey (24) and recent studies (12), indicating persistent gaps in STI knowledge. HIV/AIDS and other STI infections share a common mode of transmission and having a STI is the risk of getting HIV exposure (27). The observed discrepancy might be attributed to the disproportionate focus of public health campaigns on HIV, highlighting the need for comprehensive sexual health education that encompasses all aspects of STIs, not only HIV/AIDS. This can be addressed with community outreach programs that provide immediate access to comprehensive sexual health information and resources. Collaborating with local organizations, healthcare providers, and community leaders will help effectively reach at-risk populations. Long-term strategies should include integrating sexual health education into school curricula and providing age-appropriate information about STIs and safe practices to enhance awareness. Additionally, partnerships with NGOs and the use of digital platforms can promote the reach and impact of these initiatives.

### 4.2 Attitude

Overall, 53.5% had a good attitude toward HIV/AIDS, STI infections, and condom usage, with better attitudes toward HIV/AIDS and STI infections than condom use. Nearly half of the participants felt embarrassed to buy condoms and believed that wearing condoms reduced sexual feelings. This is supported by the findings of poor attitudes toward condom use among Myanmar migrants in Surat Thani (15) and cross-border youth in Chiang Mai (18). In addition, only 64.2% correctly identified condom use as effective in HIV prevention, reflecting the variation in social and cultural norms, personal preferences, influence of peer and partner perceptions, and access to information. Those who had less access to information regarding the effectiveness of condoms, personal preference from partners, and social norms of carrying condoms seen as promiscuity would make them less likely to view condoms favorably. The discrepancy in attitudes toward HIV/AIDS, STI infections and condom usage shows the need for tailored education and awareness campaigns that address misconceptions about condom use.

### 4.3 Sexual behaviors

Among 424 migrants, 64.6% reported sexual experience, with more males (75.5%) than females (53.8%). Condom use with regular partners was notably low (77.2% never used condoms), consistent with findings from previous studies showing 7% condom usage in Chiang Rai province (28) and 17% condom usage among migrant sex workers in Chiang Mai province (17). This could be explained by the perception of trust and intimacy leading individuals to be less likely to use condoms in emotionally close or long-term relationships. This lack of condoms used in presumed safe relationships might increase the risk of HIV/AIDS and STI infections especially when partners are unaware of each other’s HIV status or risk factors. To address this, promoting consistent condom use along with regular HIV testing should be emphasized among the sexually active community.

A total of 16 participants (5.8%) reported having non-regular partners in the past year, with 43.8% consistently using condoms. Additionally, 75% used condoms during their most recent encounter, and 50% reported having more than one non-regular partner in the last year. Compared to migrants in Ranong Province (66.7%) (29), condom usage was lower, while non-usage with non-regular partners was higher in Samut Sakhon Province (39.3%) and Patumthani Province (40.56%) (12, 25). This risky behavior favors the high risk of exposure to HIV and STI infections through associated occasional sexual partners. Targeted interventions for promoting condom use and enhancing condom accessibility are critical.

### 4.4 Preventive behaviors

91.2% of participants engaged in preventive behaviors by not having non-regular partners and always using condoms with non-regular partners or sex workers. This finding reflects a study among Myanmar migrants in Samut Sakhon province, where 85.3% engaged in protective sexual behaviors (12). Multi-structural determinants such as policies, sociocultural challenges, health factors, and risky sexual behaviors influence HIV risk (9). Factors such as age, income, HIV/AIDS awareness, lifestyle, and sexual activities are significant, highlighting the complex environment for migrants (30).

A significant association was found between the duration of stay in Chiang Mai and migrants’ preventive behaviors. Migrants staying more than 10 years showed poorer preventive behavior compared to those staying for <5 years. Longer-term migrants might become desensitized to HIV/AIDS and STI risks or face barriers in accessing ongoing prevention and education services. This could be due to factors such as socio-economic integration and cultural assimilation. Prolonged stays in other countries correlate with poor preventive behavior, especially low condom usage (30, 31). This suggests the necessity for targeted interventions for long-term migrants.

The analysis revealed that migrants in the “other” occupational category, excluding construction workers and daily workers, were significantly less likely to engage in preventive behaviors compared to “construction workers,” with an adjusted OR of 0.210 (95%Cl: 0.046–0.972). Construction sites often function as closed communities that foster peer communication and experience sharing among workers, providing a platform to promote preventive behaviors. A study among Myanmar migrant workers in Ranong province (29) found that factory workers had safer sex behaviors compared to workers in other types of jobs. Occupational differences suggest that certain types of working environments or conditions may either facilitate or hinder the adoption of prevention measures. For instance, female migrant workers in China working at entertainment venues were more likely to engage in preventive behaviors than those working in factories and restaurants (11). These differences suggest the need for targeted HIV preventive interventions. These might include workplace education programs, improved access to condoms, HIV testing and initiatives addressing specific barriers to prevention in different occupation settings. Understanding factors related to lower engagement in preventive behaviors among migrant workers in specific occupations can inform the development of more effective and context-sensitive HIV/AIDS prevention activities.

Another influencing factor affecting preventive behaviors was their attitude. Participants with a good attitude were 4.5 times more likely to engage in preventive behaviors compared to those with a poor attitude. Belief in the effectiveness of prevention methods and confidence in one’s ability to use them are crucial for behavior change (32). Additionally, activities that increase migrants’ confidence, such as practical demonstrations on proper condom use and easy access to HIV testing, and counseling, will further encourage preventive behaviors.

The study findings show that migrants have low STI knowledge and poor attitudes toward condom use, with preventive behavior being particularly rare among those who have lived for over 10 years and work in non-construction jobs. To support the Sustainable Development Goals of eliminating HIV and STIs by 2030 (33), it is recommended to expand culturally sensitive, tailored health education programs, provide accessible HIV early testing services, and strengthen collaboration between governmental and non-governmental organizations to improve service delivery and reduce risk factors among migrants.

While previous research had identified socio-demographic factors such as gender and marital status (29, 31, 34), education level (17), and income-expense balance (12) as influencing factors on preventive behaviors among migrants, this study found no such association. This is consistent with findings from studies in Bangkok and the 2010 migrant survey, where gender, marital status (35, 36) and education level (36) were also not related to HIV preventive behaviors among migrant workers. Those could be explained by differences in demographic composition and socio-economic context of the study populations which might vary in behaviors and attitudes subsequently. Moreover, preventive behaviors might vary significantly across the different sub-populations of migrants, such as sex workers and migrants living with HIV, where the study population in this study was the general migrant population. The diversity within the migrant population likely contributes to the complexity of behaviors and determinants, and indicating the targeted interventions could be more effective.

### 4.5 Limitations

Bias might arise from hospital-based data collection and self-reported information, potentially influenced by social desirability bias or recall issues. The convenience sampling method and the focus on Chiang Mai province limit generalizability to broader migrant populations and other regions. The cross-sectional study design could not draw causality conclusions from the analysis. A 17.9% non-response rate on sensitive behavior questions, particularly among certain demographics, introduces potential bias, possibly affecting the interpretation of preventive behaviors.

## 5 Conclusion

The study highlighted significant gaps in STI knowledge and attitudes toward condom usage among Myanmar migrants in Chiang Mai province, Thailand. Despite a reasonable level of HIV/AIDS knowledge, misconceptions about transmission and prevention persist, particularly regarding condom use and attitudes toward people living with HIV/AIDS.

## Data Availability

The raw data supporting the conclusions of this article will be made available by the authors, without undue reservation.
