# Supplementary material for: Knowledge, attitude, and preventive behaviors related to HIV/AIDS and sexually transmitted infections among Myanmar migrants in Chiang Mai province, Thailand
Source: Front Public Health. 2024 Nov 27;12:1478592. doi: 10.3389/fpubh.2024.1478592 (PMC11631854; doi:10.3389/fpubh.2024.1478592)
Supplement: Supplementary file 1 [file Table_1.DOCX]

Supplementary table 1. Sexual behaviors related to HIV/AIDS and STIs (n=424)

| **Sexual Behaviors** | **Male** | |  | **Female** | |  | **Total** | |
| --- | --- | --- | --- | --- | --- | --- | --- | --- |
|  | **n** | **%** |  | **n** | **%** |  | **n** | **%** |
| Age at first having sex (n=274) |  |  |  |  |  |  |  |  |
| ≤15 yrs. | 13 | 8.1 |  | 5 | 4.4 |  | 18 | 6.6 |
| 16-20 yrs. | 112 | 70.0 |  | 76 | 66.7 |  | 188 | 68.6 |
| 21-25 yrs. | 24 | 15.0 |  | 19 | 16.7 |  | 43 | 15.7 |
| 26-30 yrs. | 8 | 5.0 |  | 10 | 8.8 |  | 18 | 6.6 |
| >30 yrs. | 1 | 0.6 |  | 2 | 1.8 |  | 3 | 1.1 |
| Don't remember | 1 | 0.6 |  | 0 | 0.0 |  | 1 | 0.4 |
| No response | 1 | 0.6 |  | 2 | 1.8 |  | 3 | 1.1 |
| Kind of sexual activity (multiple choices) |  |  |  |  |  |  |  |  |
| Vaginal Sex | 144 | 86.2 |  | 103 | 89.6 |  | 247 | 87.6 |
| Anal Sex | 2 | 1.2 |  | 1 | 0.9 |  | 3 | 1.1 |
| Oral Sex | 6 | 3.6 |  | 0 | 0.0 |  | 6 | 2.1 |
| Other | 1 | 0.6 |  | 1 | 0.9 |  | 2 | 0.7 |
| No Response | 14 | 8.4 |  | 10 | 8.7 |  | 24 | 8.5 |
| Regular partner status |  |  |  |  |  |  |  |  |
| Yes, not living together | 11 | 6.9 |  | 14 | 12.3 |  | 25 | 9.1 |
| Yes, living together | 108 | 67.5 |  | 82 | 71.9 |  | 190 | 69.3 |
| No | 30 | 18.8 |  | 13 | 11.4 |  | 43 | 15.7 |
| No response | 11 | 6.9 |  | 5 | 4.4 |  | 16 | 5.8 |
| Condom use with regular partner (n=215) |  |  |  |  |  |  |  |  |
| Always | 1 | 0.8 |  | 4 | 4.2 |  | 5 | 2.3 |
| Most of the times | 1 | 0.8 |  | 2 | 2.1 |  | 3 | 1.4 |
| Sometimes | 22 | 18.5 |  | 5 | 5.2 |  | 27 | 12.6 |
| Never | 89 | 74.8 |  | 77 | 80.2 |  | 166 | 77.2 |
| No sexual activity | 4 | 3.4 |  | 3 | 3.1 |  | 7 | 3.3 |
| No response | 2 | 1.7 |  | 5 | 5.2 |  | 7 | 3.3 |
| Have non-regular partner including sex workers in the last 12 months (n=274) |  |  |  |  |  |  |  |  |
| Yes | 11 | 6.9 |  | 5 | 4.4 |  | 16 | 5.8 |
| No | 138 | 86.3 |  | 104 | 91.2 |  | 242 | 88.3 |
| No response | 11 | 6.9 |  | 5 | 4.4 |  | 16 | 5.8 |
| Number of non-regular partner (n=16) |  |  |  |  |  |  |  |  |
| 1 | 2 | 18.2 |  | 2 | 40.0 |  | 4 | 25.0 |
| >1 | 6 | 18.2 |  | 2 | 40.0 |  | 8 | 50.0 |
| No response | 3 | 27.3 |  | 1 | 20.0 |  | 4 | 25.0 |
| Condom use with non-regular partner (n=16) |  |  |  |  |  |  |  |  |
| Always | 5 | 45.5 |  | 2 | 40.0 |  | 7 | 43.8 |
| Most of the time | 0 | 0.0 |  | 1 | 20.0 |  | 1 | 6.3 |
| Sometimes | 5 | 45.5 |  | 0 | 0.0 |  | 5 | 31.3 |
| Never in 12 months | 1 | 9.1 |  | 1 | 20.0 |  | 2 | 12.5 |
| No response | 0 | 0.0 |  | 1 | 20.0 |  | 1 | 6.3 |
| Condom use with last non-regular partner (n=16) |  |  |  |  |  |  |  |  |
| Yes | 10 | 90.9 |  | 2 | 40.0 |  | 12 | 75.0 |
| No | 1 | 9.1 |  | 2 | 40.0 |  | 3 | 18.8 |
| No response | 0 | 0.0 |  | 1 | 20.0 |  | 1 | 6.3 |
| Visit to sex workers in the last 6 months (n=274) |  |  |  |  |  |  |  |  |
| Yes | 4 | 2.5 |  | 0 | 0.0 |  | 4 | 1.5 |
| No | 144 | 90.0 |  | 109 | 95.6 |  | 253 | 92.3 |
| No response | 12 | 7.5 |  | 5 | 4.4 |  | 17 | 6.2 |
| Condom use with sex workers in the past 6 months (n=4) |  |  |  |  |  |  |  |  |
| Always | 3 | 75.0 |  | 0 | 0.0 |  | 3 | 75.0 |
| Sometimes | 1 | 25.0 |  | 0 | 0.0 |  | 1 | 25.0 |
| Condom use with last sex workers |  |  |  |  |  |  |  |  |
| Yes | 4 | 100.0 |  | 0 | 0.0 |  | 4 | 100.0 |
| Ever had STIs in the last 12 months (n=274) |  |  |  |  |  |  |  |  |
| Yes | 2 | 1.3 |  | 1 | 0.9 |  | 3 | 1.1 |
| No | 141 | 88.1 |  | 92 | 80.7 |  | 233 | 85 |
| Don't remember | 6 | 3.8 |  | 13 | 11.4 |  | 19 | 6.9 |
| No response | 11 | 6.9 |  | 8 | 7.0 |  | 19 | 6.9 |
| Name of the last STIs (n=3) |  |  |  |  |  |  |  |  |
| Gonorrhea | 2 | 100.0 |  | 0 | 0.0 |  | 2 | 66.7 |
| No response | 0 | 0.0 |  | 1 | 100.0 |  | 1 | 33.3 |
| Treatment for last STIs |  |  |  |  |  |  |  |  |
| Self-care at home | 1 | 50.0 |  | 1 | 100.0 |  | 2 | 66.7 |
| Government hospital | 1 | 50.0 |  | 0 | 0.0 |  | 1 | 33.3 |
